# User Acceptance of Computerized Cognitive Behavioral Therapy for Depression: Systematic Review

**DOI:** 10.2196/jmir.7662

**Published:** 2017-09-13

**Authors:** Theresia Rost, Janine Stein, Margrit Löbner, Anette Kersting, Claudia Luck-Sikorski, Steffi G Riedel-Heller

**Affiliations:** ^1^ Institute of Social Medicine, Occupational Health and Public Health Medical Faculty University of Leipzig Leipzig Germany; ^2^ Department of Psychosomatic Medicine University Medical Centre University of Leipzig Leipzig Germany; ^3^ Mental Health and Psychotherapy Stiftung Rehabilitation Heidelberg University of Applied Health Services Gera Germany; ^4^ Integrated Research and Treatment Center (IFB) AdiposityDiseases University Hospital Leipzig Germany

**Keywords:** computerized cognitive behavior therapy, depression, user acceptance, systematic review, review, behavior therapy, depressive disorder

## Abstract

**Background:**

Computerized cognitive behavioral therapy (cCBT) has been proven to be effective in depression care. Moreover, cCBT packages are becoming increasingly popular. A central aspect concerning the take-up and success of any treatment is its user acceptance.

**Objective:**

The aim of this study was to update and expand on earlier work on user acceptance of cCBT for depression.

**Methods:**

This paper systematically reviewed quantitative and qualitative studies regarding the user acceptance of cCBT for depression. The initial search was conducted in January 2016 and involved the following databases: Web of Science, PubMed, the Cochrane Library, and PsycINFO. Studies were retained if they described the explicit examination of the user acceptance, experiences, or satisfaction related to a cCBT intervention, if they reported depression as a primary outcome, and if they were published in German or English from July 2007 onward.

**Results:**

A total of 1736 studies were identified, of which 29 studies were eligible for review. User acceptance was operationalized and analyzed very heterogeneously. Eight studies reported a very high level of acceptance, 17 indicated a high level of acceptance, and one study showed a moderate level of acceptance. Two qualitative studies considered the positive and negative aspects concerning the user acceptance of cCBT. However, a substantial proportion of reviewed studies revealed several methodical shortcomings.

**Conclusions:**

In general, people experience cCBT for depression as predominantly positive, which supports the potential role of these innovative treatments. However, methodological challenges do exist in terms of defining user acceptance, clear operationalization of concepts, and measurement.

## Introduction

Depressive disorders are among the most common and serious mental illnesses [1]. Globally, 350 million people of all ages are estimated to suffer from depression. If depressive disorders are detected at an early stage, they are highly treatable in the majority of cases [2]. There are known effective psychological treatments, for example, cognitive behavioral therapy (CBT) [3]. However, individuals suffering from depression often find themselves confronted with barriers to receiving appropriate care such as social stigma associated with mental disorders, long waiting times, or the logistical difficulties of appearing in person for treatment [4,5]. For these reasons, computerized programs present an innovative approach to improving access to psychological treatments for depression. There is evidence that computerized cognitive behavioral therapy (cCBT) is effective in the treatment of various mental disorders, including depression [6-10]. There are a number of advantages that are associated with cCBT such as anonymity, wide availability, or location-independent and around-the-clock access [9,11]. Well-known cCBT programs such as Beating The Blues and MoodGYM have been shown to provide a promising option for the treatment of mental health problems [9,12,13]. A prerequisite for cCBT programs to be effective is its user acceptance, as the implementation of an innovative intervention such as cCBT can be affected negatively because of individuals being unwilling to use it. For example, the absence of a contact person and the resulting anonymity can have a negative impact on the user’s motivation to start or keep up with a cCBT program. Therefore, it is of utmost importance to consider user acceptance when developing and implementing a cCBT program for the treatment of depression.

The concept of user acceptance arose as a key term in the scientific discourse. Definitions of the term differ widely depending on the intended use [14]. One of the most popular approaches is the technology acceptance model (TAM) developed by Davis [15]. TAM illustrates user acceptance determined by two factors: perceived usefulness and perceived ease of use. According to Davis [15], both have a significant impact on a person’s attitude toward using a new technology. Kollmann [16] and Rogers [17] went one step further and combined different phases in their acceptance models. Therefore, the user passes through phases from getting to know a new technology, to forming an attitude toward it, to a decision whether to use or not to the confirmation of the decision. On this basis, user acceptance can be defined as the willingness of individuals to employ information technology for the tasks it is designed to support, the realization, and approval of the decision to employ. All of these models have one thing in common: user acceptance is considered to be a process beginning with an attitude toward the innovation and developing into satisfaction with the innovation; it is not an instantaneous act. Accordingly, we have conceived acceptance as the act of accepting, experiencing, and being satisfied.

Since the emergence of the first cCBT programs, there have been a number of reviews addressing the user acceptance of cCBT; however, they have utilized different approaches. In their reviews, Titov [18], Andrews et al [8], and Vallury et al [19] focused broadly on effectiveness and user acceptance of cCBT for several mental disorders, including depression and anxiety disorders. Waller and Gilbody [20] reviewed quantitative and qualitative studies examining adverse consequences, accessibility, and acceptability of cCBT programs for treating anxiety and depression. However, Kaltenthaler et al [21] provide the only review with a very comprehensive and focused insight into the user acceptance of cCBT for depression, including research up to June 2007. They systematically reviewed sources of information on acceptability to patients of cCBT for depression. As a result, they documented several studies reporting positive expectancies and high satisfaction in routine care cCBT services for those completing the treatment and argued that studies should reveal more detailed information on patient recruitment methods, dropout rates, and reasons for dropping out. Furthermore, they drafted well-designed surveys and qualitative studies included alongside trials to determine levels of patient acceptability as implications for further research.

On this basis, we provide a systematic overview on user acceptance of cCBT for depression over the last 10 years and widen the perspective to include the notion that the process of user acceptance spans a number of phases, including accepting, experiencing, and being satisfied with cCBT. We intend to answer the following research questions: (1) which measures were used to examine the user acceptance of cCBT for depression? and (2) to what degree do users accept cCBT for depression?

## Methods

This systematic review was conducted according to guidelines from the Preferred Reporting Items for Systematic Reviews and Meta-Analyses (PRISMA) statement [22]. On the basis of the PICO (Patient, problem, or population; Intervention; Comparison, control, or comparator; Outcome) approach and the review by Kaltenthaler et al [21], the criteria for inclusion were as follows.

### Eligibility Criteria

#### Population

Since cCBT programs may be also designed for people not undergoing medical treatment, we decided to widen the focus on people with or without medical attention. Thus, studies with participants of all ages with a diagnosis of depression of all degrees of severity were regarded as eligible for inclusion in this review.

#### Interventions

All cCBT interventions and their subtypes (eg, mindfulness-based cognitive therapy and behavioral activation) delivered alone or as part of a package of care via the Internet were taken into consideration.

#### Comparison

Randomized controlled trials (RCTs), nonrandomized comparative trials, noncomparative trials, and qualitative studies published from July 2007 to January 2016 were included.

#### Outcome

Studies were included if they reported on the following: data on user acceptance in terms of acceptability, satisfaction, or experiences concerning cCBT; studies with depression as a primary outcome; and studies providing information on study design and measures, including a description of the delivered treatment and the sample including the number, age, and sex of participants. Studies were excluded if they were not reported in English or German or if they were single case reports.

### Search

The search for relevant literature was conducted in four bibliographic databases from July 1, 2007 to January 31, 2016, which are as follows: Web of Science, PubMed, the Cochrane Library, and PsycINFO. Furthermore, the bibliographies of identified papers were searched to identify other potentially eligible papers. Since studies about user acceptance emanate from a young research area, it is conceivable that many studies measure or report about it via proxy indices, which was considered in the search strategy of this study. Considering British and American spelling, a search strategy combining the following search terms was used to ensure complete coverage of studies: Concept 1 (“internet” OR “web” OR “DVD” OR “CD-ROM” OR “online” OR “computer*” OR “e-health” OR “electronic” OR “program” OR “programme”) AND Concept 2 (“CCBT” OR “CBT” OR “cognitive therapy” OR “behavior therapy” OR “behavioral therapy” OR “behavioural therapy” OR “behaviour therapy”) AND Concept 3 (“accept*” OR “satisfaction” OR “adherence” OR “compliance” OR “take up rates” OR “patient dropout rates” OR “reasons for dropout” OR “patient drop-out rates” OR “reasons for drop-out”) AND Concept 4 (“depress*” OR “dysthym*” OR “mood disorder” OR “affective disorder” OR “melancholia”).

### Study Selection

After removing duplicates identified in databases and reference lists, titles and abstracts of the texts were scanned to examine indications for meeting the inclusion criteria. For all remaining papers that deemed relevant, the full text was reviewed. All information from the included studies was gathered by one reviewer and checked by a second.

### Data Collection Process

We extracted information on the characteristics of the program, as well as information on the study design, the setting, the ways of recruitment, the sample, dropout and completion rates, and, if available, reasons for dropout.

### Synthesis of Results

To allow a better comparability, we transferred the results into levels of acceptance that range from low (−−) to moderate (−) to high (+) to very high (++). The levels follow the results reported in percentage and scale values that were assigned to quartiles. Therefore, results ranging between 0% and 25% were assigned to *low*, between 26% and 50% to *moderate*, 51% and 75% to *high*, and 76% and 100% to *very high*. This also applies equivalently to scale values. For example, Danaher et al [23] used a 4-point scale for the elicitation of satisfaction (1=not at all satisfied, 4=very satisfied), for which the quartiles are as follows: 1 to 1.75 (low), 1.76 to 2.5 (moderate), 2.6 to 3.25 (high), and 3.26 to 4 (very high). The study reported mean scores of 3.3 (satisfaction with features of the program) and 3.4 (helpfulness of personal coach calls). Thus, the level of acceptance can be described as *very high*.

If there were considerations of positive and negatives aspects concerning the user acceptance of cCBT, they were characterized (~).

## Results

### Study Selection

**Figure 1:**
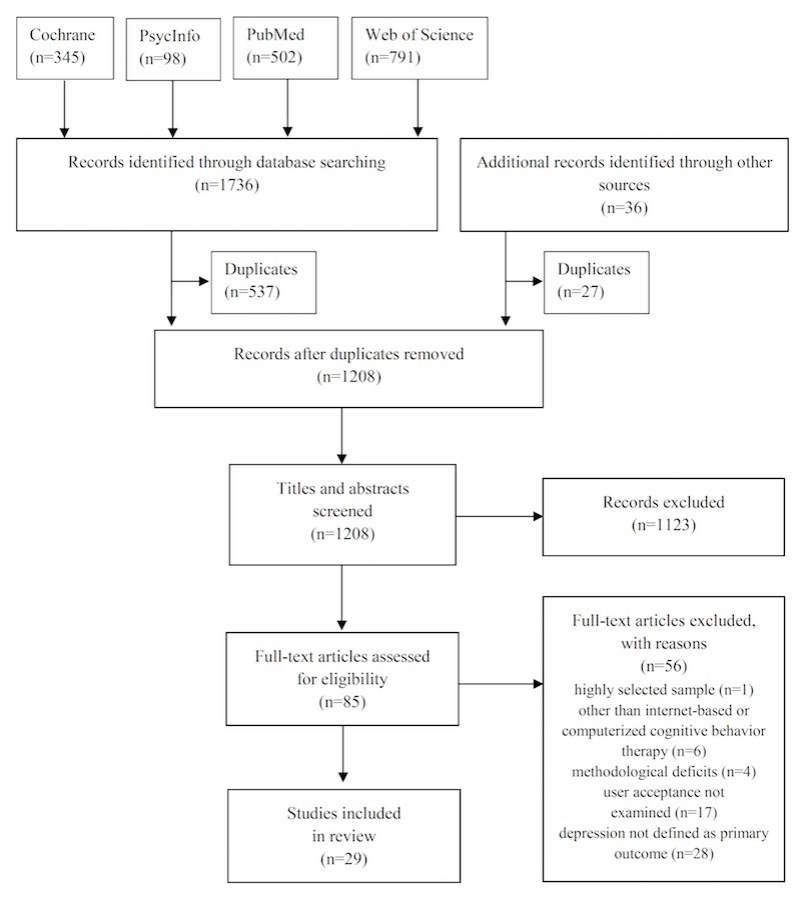
Preferred Reporting Items for Systematic Reviews and Meta-Analyses (PRISMA) flowchart of the study selection and eligibility process.

As shown in Figure 1, a total of 1736 potentially relevant papers were identified through database searching; 36 additional papers were identified from reference lists. After removing a total of 564 duplicates, 1208 papers were screened based on their titles and abstracts. A total of 1123 publications were excluded because they did not fulfill the inclusion criteria. Hence, the remaining 85 full-text papers were assessed for eligibility. Of those, 56 publications were excluded mainly because depression was not defined as a primary outcome (n=28), and user acceptance was not examined as described in their titles or abstracts (n=17). Furthermore, one study could not be taken into consideration because of a highly selected sample comprising caregivers of anorexia nervosa patients. Finally, we included 29 studies for further analysis.

### Study Characteristics

Objects of investigation were several cCBT programs, including “MoodGYM” and “Beating the Blues” that were examined most commonly. Table 1 presents further information on the characteristics of the cCBT programs. As Multimedia Appendix 1 shows, 16 of included studies were RCTs, 8 were noncomparative trials, 3 were qualitative studies, and 2 were comparative but nonrandomized trials. Five of the studies had a special feature: three studies made a comparison between guided and unguided programs [24-26] and the remaining two studies compared two programs [27,28].

**Table 1:** Characteristics of the computerized cognitive behavioral therapy (cCBT) programs.

Author, year, country	Description of the program	Support (nontherapeutic support; therapist support)
Ahmedani et al, 2015, United States [29]	iCBT^a^ program (no name), brief tailored mobile health inter­ven­tion, based on a combination of motivational interviewing and CBT^b^ models	yes; no
Berger et al, 2011, Switzer­land [24]	Deprexis, a self-help program comprising 10 content modules and a summary module covering a variety of therapeutic content that is broadly consistent with a cognitive behavioral model	no; yes (partici­pants ran­domized in guided self-help condition received email con­tact with a therapist)
	IG^c^ I: unguided self-help condition	
	IG II: guided self-help condition	
Berman et al, 2014, United States [30]	ePST, a 6-session, stand-alone multimedia, interactive, computer-based problem-solving treatment	yes; no
Boescho­ten et al, 2012, The Nether­lands [31]	cCBT^d^ program (no name), based on the original “problem-solving therapy,” adjusted for multiple sclerosis patients with comorbid depression and comprising 5 modules containing text, exercise, and examples	yes; no
Burns et al, 2011, United States [32]	Mobilyze!, an 8-week mobile phone– and Internet-based inter­vention for depression	yes; yes
Cartreine et al, 2012, United States [33]	ePST, a 6-session, stand-alone multimedia, interactive, computer-based problem-solving treatment	not reported
Choi et al, 2012, Australia [34]	The Brighten Your Mood Program, a cultural adapted version of the Sadness Program comprising 6 lessons	yes; no
Danaher et al, 2013, United States [23]	MomMoodBooster *,* an interactive guided Web-based intervention for postpartum depression comprising 6 sessions	yes
de Graaf et al, 2009, The Nether­lands [35]	Colour Your Life, a Web-based multimedia, interactive, self-help cCBT program for depression based on the Dutch version “Coping With Depression course” and comprises 8 weekly sessions	no; only partici­pants who got the inter­vention + TAU
	Colour Your Life + TAU^e^	
Dear et al, 2013, Australia [36]	Managing Your Mood, a structured 5-lesson Web-based intervention that encourages participants to learn and practice core CBT psychological skills	yes
Dimidjian et al, 2014, United States [37]	Mindful Mood Balance, a Web-based, 8-session self-administered platform	not reported

Geraedts et al, 2015, The Nether­lands [38]	Happy@Work, a brief 6-lesson Internet intervention based on problem-solving treatment, cognitive therapy, and a guideline for employees to help them prevent work-related stress	yes
Gerhards et al, 2011, The Nether­lands [39]	Colour Your Life *,* a Web-based multimedia, interactive computer program for depression comprising 8 weekly sessions and a 9th booster session	no; no
Hind et al, 2010, United Kingdom [27]	Beating the Blues, an interactive computer program with 8 modules for the treatment of depressive and anxiety disorders	yes
	MoodGYM, a freeware cCBT program comprising 5 modules	
	IG I: Beating the Blues	
	IG II: MoodGYM	
Høifødt et al, 2013, Norway [12]	MoodGYM, a Web-based program containing 5 modules comprising written information, animations, interactive exercises, and quizzes	no; yes
Kay-Lambkin et al, 2011, Australia [40]	SHADE, a clinician-assisted computer-based psychological treatment comprising 10 sessions and delivered on DVD	yes
Knowles et al, 2015, United Kingdom [28]	MoodGYM, a Web-based program containing 5 modules comprising written information, animations, interactive exercises, and quizzes	no
	Beating the Blues, an interactive computer program with 8 modules for the treatment of depressive and anxiety disorders	
Kok et al, 2014, The Nether­lands [41]	Depressionfree, comprising Internet-based preventive cognitive therapy with 8 modules, telephone-delivered psychotherapy and mood monitoring	yes; yes
	Depressionfree + TAU	
Lintvedt et al, 2013, Norway [42]	MoodGYM, a self-help program based on principles of CBT, interpersonal therapy, and relaxation techniques comprising 5 modules	no; no
	BluePages provides evidence-based information about depression	
Lucassen et al, 2014, New Zealand [43]	Rainbow SPARX, an interactive fantasy game comprising 7 modules designed to deliver CBT for the treatment of clinically significant depression; customized for sexual minority youth	yes
McMur­chie et al, 2013, United Kingdom [44]	Beating the Blues, an interactive computer program with 8 modules for the treatment of depressive and anxiety disorders	yes; no
	Beating the Blues + TAU	
Merry et al, 2012, New Zealand [45]	SPARX, an interactive fantasy game comprising 7 modules designed to deliver CBT for the treatment of clinically significant depression	no; no
O'Mahen et al, 2013, United Kingdom [46]	Postnatal Internet-based behavioral activation (iBA^f^), adapted for postnatal Web-based delivery from the manual developed for behavioral activation, comprising 11 weekly sessions	Access to Netmums’ general depression chat room monitored by parent supporters and specialist health visitors
	Postnatal iBA + TAU	
Perini et al, 2009, Australia [47]	The Sadness Program, a cCBT comprising 6 Web-based lessons, homework assignments, participation in an online discussion forum, and regular email contact with a mental health clinician	yes; yes
Richards and Timulak, 2013, Ireland [25]	Beating the Blues, an interactive computer program with 8 modules for the treatment of depressive and anxiety disorders	no; only participants who were treated with intervention II got additional support from a therapist
	IG I: Self-administered Beating the Blues	
	IG II: Therapist-delivered Beating the Blues	
Schneider et al, 2014, United Kingdom [48]	MoodGYM, a Web-based program containing 5 modules comprising written information, animations, interactive exercises, and quizzes	yes; no
Sheeber et al, 2012, United States [49]	Mom-Net program, an 8-session, Internet-facilitated CBT treatment for subthreshold and full syndrome depression, tailored to mothers of young children; the content foundation for the program was the Coping With Depression course	yes
	IG I: Internet-facilitated intervention	
	IG II: Delayed intervention or facilitated TAU	
Stasiak et al, 2014, New Zealand [50]	The journey, a cCBT with 7 modules of well-established core cognitive behavioral therapy techniques.	yes; no
Titov et al, 2010, Australia [26]	The Sadness Program, a cCBT program comprising 6 Web-based lessons, printable summary and homework assignments, automatic emails, and additional resource documents	yes; yes
	IG I: Technician-assisted group	
	IG II: Clinician-assisted group	

^a^iCBT: Internet-based cognitive behavioral therapy.

^b^CBT: cognitive behavioral therapy.

^c^IG: intervention group.

^d^cCBT: computerized cognitive behavioral therapy.

^e^TAU: treatment-as-usual.

^f^iBA: Internet-based behavioral activation.

### Measures of Acceptance

As illustrated in Multimedia Appendix 2, the studies made use of several measures to examine the user acceptance of cCBT. The large majority of studies (n=25) used direct measures such as questionnaires or qualitative methods; two studies used indirect measures such as take-up rates, completion rates, or dropout rates; and two studies used a combination of direct and indirect measures. Of those using direct measures, four employed qualitative methods, five made use of well-established questionnaires, and 16 used study-specific questionnaires. These study-specific questionnaires varied substantially in their level of complexity. For example, Dear et al [36] ascertained the user acceptance of cCBT through 2 questions: (1) would you recommend the program to a friend? and (2) was the program worth your time? Berman et al [30] and Cartreine et al [33] employed the Acceptability of Self-Guided Treatment Questionnaire (AST) with 16 statements to be rated on a 7-point scale. The majority of study-specific developed questionnaires were left unspecified or roughly outlined (see Multimedia Appendix 2).

There were four studies that ascertained take-up, dropout, or completion rates as a means of assessing the user acceptance of cCBT. As Multimedia Appendix 3 shows, plenty of studies provided dropout and completion rates by default. Therefore, 17 studies revealed information about program completion, three reported dropout rates, seven trials commented on both, and two studies did not give any information about completion or dropout rates. For those reporting on rates for completing the entire program, the mean percentage of completion was 67.17% (standard deviation [SD] 20.29) with a range of 26.7% to 100%. With regard to the trials that compared guided with unguided programs, highly varying completion rates have been reported. Whereas Berger et al [24] documented that 36% of participants in the unguided self-help condition and 56% of participants in the guided self-help condition completed the entire program, Richards and Timulak [25] reported a completion rate of 16.28% in the unguided condition and 8.11% in the guided condition.

For the studies reporting on dropout rates, the mean percentage of dropout rates was 31.5% (SD 19.49), with a range of 0% to 63%. Twelve trials listed reasons for dropout. The most commonly stated reasons were a lack of time (n=6), technical difficulties, or computer-related problems (n=4), or participants experiencing the treatment as inconvenient (n=4). Since the trials differed in terms of study design, the extent of disclosure, and definitions of dropout and completion, it was difficult to draw comparisons between them regarding completion and dropout rates. Moreover, four studies documented take-up rates as follows: 83.3% [39], 56.9% [44], 97% [40], and 39% [12].

### User Acceptance: Acceptability, Satisfaction, and Experiences

Multimedia Appendix 2 shows the results of all eligible studies regarding the user acceptance in terms of acceptability, satisfaction, and experiences with cCBT for depression. As shown here, results were primarily reported descriptively (eg, by reference to rated statements or responses to questions in a qualitative dimension) irrespective of whether the study type was quantitative or qualitative. According to the levels adapted by the author, eight studies (28%) reported a very high level of acceptance [23,26,36,43,45,47-49], 17 studies (59%) indicated a high level of acceptance [12,24,25,29-35,38,40-42,44,46,50], and one study (3%) showed a moderate level of acceptance [27]. No study showed a low level of user acceptance. One study (3%) gave conflicting information [37], which is why an allocation to a level of acceptance was not possible.

Two qualitative studies (7%) referred to considerations of positive and negative aspects concerning the user acceptance of cCBT. Specifically, Gerhards et al [39] and Knowles et al [28] focused on differentiated perceptions of cCBT that they extracted from qualitative interviews with participants. Gerhards et al [39] described the main barriers to be a lack of identification with cCBT, an absence of support to adhere to the program to gain deeper understanding, and inadequate computer and Internet skills. Motivators included the opportunity to use the program independent of time of day and location and added support as an improvement with regard to adherence and the course content. Knowles et al [28] showed that the same aspects of cCBT could be perceived positively and negatively, depending on the participant’s experience and preference. For example, anonymity was associated with reduced pressure as compared with being face-to-face; however, it was also experienced as isolating and enhancing the feeling of loneliness. Similarly, flexibility was experienced as positive because patients are afforded a high degree of control but also as negative because the program can be seen as to be easy to avoid and difficult to sustain.

One special feature of three of the studies is the comparison of guided and unguided programs [24-26]. Whereas Titov et al [26] did not ascertain any differences concerning the satisfaction between the clinician-assisted cCBT and the technician-assisted cCBT, Berger et al [24] found evidence that participants in the guided condition were a little more satisfied than those in the unguided condition. Richards and Timulak [25] documented that most participants in both groups found the treatment helpful, even though there was a nonsignificant trend showing that participants in the unguided condition found the treatment easy to use and were more likely to report lasting effects than participants in the guided condition.

Although results give evidence of cCBT for depression being highly accepted, it should be noted that several studies do not give an exact definition of their object of investigation. As a consequence, the studies’ stated objective is not in accordance with the measures that were used to examine the user acceptance of cCBT. These questionnaires did not refer explicitly to acceptability, satisfaction, or experiences but target related constructs such as ease of use [25,49], usefulness [41,42], or credibility or expectancy [26].

## Discussion

### Summary of Evidence

We intended to conduct a comprehensive review of studies regarding the user acceptance of cCBT for depression, updating the findings of Kaltenthaler et al [21] who aimed to assess studies regarding the acceptability to patients of cCBT for depression. Because user acceptance of a treatment may be a determinant for individuals to start and adhere to cCBT, the objective of this paper was to systematically evaluate studies that refer to the user acceptance of cCBT for depression in terms of acceptability, satisfaction, and experiences.

Corresponding with the findings of Kaltenthaler et al [21], the majority of the 29 reviewed studies reveal high or very high levels of user acceptance of cCBT programs. In addition to scientifically proven effectiveness of cCBT for depression [6-10], this result indicates a positive prognosis for future usage of cCBT programs.

When examining the user acceptance of cCBT for depression, most studies employed direct measures. Only a few studies made use of only indirect measures and consulted take-up, dropout, or completion rates for examining the user acceptance of cCBT [37,40,44]. In light of the fact that there was no information provided as to why participants do not start or continue a program, the validity of take-up, dropout, and completion rates is limited. Therefore, reasons that were not associated with the quality and appeal of a cCBT program, such as technical problems (eg, incompatible graphics software), personal reasons (eg, a lack of motivation because of a medical condition), or circumstances of research (eg, availability of an incentive) can lead to misinterpretations in terms of user acceptance. Kaltenthaler et al [21] had similar objections and concluded that the refusal to take part in a study regarding cCBT may show reluctance to enter a trial, rather than a dislike of cCBT. To examine the reasons why eligible persons who consented to participate in cCBT chose not to begin or drop out of the program, qualitative research efforts should be developed similar to those of Gerhards et al [39].

In general, the reported take-up rates for cCBT programs were wide ranging, making it difficult to draw comparisons with take-up rates for face-to-face CBT. However, the majority of studies reported dropout rates that are comparable with those reported for face-to-face CBT. In the RCT by Ekeblad et al [51], there was a dropout rate of 40%. Hans and Hiller [52] published a meta-analysis in which a dropout rate of 24.63% with a range from 0% to 68% was reported. Thimm and Antonsen [53] conducted a trial that revealed a dropout rate of 17.5%.

Upon closer examination of the reviewed studies, a number of methodological inaccuracies become apparent. Often no precise distinctions were made regarding the definition of acceptance, operationalization, and presentation of results. As a consequence, terms such as acceptance or acceptability, satisfaction, and usability were used interchangeably, although they can have different meanings [12,23,26,34,36,42,47]. Moreover, measures that did not correspond to the object of investigation were used [12,23,26,36,37,41,42]. These findings imply that research on acceptance is reflective of the fact that this is still a young field and, hence, there is a lack of precise definitions and adequate quantitative and qualitative measures, as these can only be realized over time.

These theoretical considerations are central to Kollmann [16] and Roger [17] who analyzed the construct of acceptance. Kollmann [16] defines acceptance as a combination of the inner reflection and the expectation formation (level of attitude), an adoption of the innovative product (level of action), and a voluntary problem-centered use of it (level of usage). This corresponds with the reflections of Rogers [17] who developed a model of stages in the innovation-decision process, which posits that individuals pass through an innovation-decision process, starting from first knowledge of an innovation such as cCBT, to forming an attitude toward the innovation, to deciding to adopt or reject, to implementing the new idea, and finally to confirming of this decision [17]. Thus, a person’s decision about the engagement with an innovative computerized treatment is not an instantaneous act; rather, it is a process that occurs over time, comprising a series of actions and decisions [17]. The examination of all these levels or stages culminating in acceptance over time requires a number of well-matched measures that can be employed longitudinally.

The studies that made a comparison between guided and unguided cCBT programs regarding user acceptance revealed highly diverse results [24-26]. Further research is needed to shed more light on the user acceptance of guided and unguided cCBT programs.

Differentiated user perceptions of cCBT were central in the presentation of qualitative results by Gerhards et al [39] and Knowles et al [28]. Both gained deeper insights into the perspective of participants toward the user acceptance of cCBT for depression with the help of semistructured interviews. The findings were expressed in terms of motivators and barriers, as well as strengths and weaknesses. Therefore, these qualitative findings extend the knowledge gained in quantitative studies by providing cross-connections of participants’ views and comprehensive insights in their experiences of these innovative treatments.

### Strengths and Limitations

To our knowledge, this is the first review updating the state of the art regarding the user acceptance of cCBT for depression since Kaltenthaler et al [21] published their review about the patient acceptability of cCBT for depression in 2008. The major strength of this review is the comprehensive insight into the state of research regarding the user acceptance of cCBT by looking at various study types that give information about different approaches to ascertain the user acceptance of cCBT for depression.

There are a number of limitations to this review. The results of the studies provide a good overview of the user acceptance of cCBT for depression; however, they differ considerably in design, including sample characteristics, program features, and the condition under which treatment was offered. For example, four studies gave information on the user acceptance of cCBT reporting only on those participants who completed the treatment [29,32,33,50], whereas 18 studies analyzed data on user acceptance of cCBT also from noncompleters [12,24,26-28,30,31,34,35,37-40,43,44,47-49]. Seven studies did not provide any information as to whether completers or noncompleters or both had been included in analyses regarding the user acceptance of cCBT [23,25,36,41,42,45,46]. These different approaches to the consideration of participants for analysis make the studies difficult to compare.

There had been considerations to assess the quality of the studies formally. Since we included various study types ranging from RCTs to comparative trials to qualitative studies (see Multimedia Appendix 1), it was difficult to scrutinize them. Therefore, we decided to waive a quality assessment.

Furthermore, research on user acceptance is vulnerable to a selection bias because the process of accepting may already begin “before” using an innovative treatment, which means that people who have reservations regarding cCBT for depression may do not get involved in the first place. Moreover, it remains unknown if the refusal to participate in a study originates from reservations regarding cCBT or research itself. At the same time, research aspects may have an opposite unintended consequence; the program may encourage participation and adherence simply because it is being researched. Furthermore, user acceptance of cCBT for depression may be influenced by aspects associated with the user’s medical condition. Thus, depressive mood, a loss of energy and drive as characteristics of depressive disorders may affect the motivation to start or adhere to cCBT. In addition to these aspects, the severity of symptoms and possible comorbidities are difficult to examine in terms of user acceptance.

### Conclusions and Implications for Further Research

In conclusion, users of cCBT for depression experience the treatment as predominantly positive, which supports the potential benefit of innovative treatments such as cCBT. The preferred measures for examining the user acceptance in terms of acceptability, satisfaction, and experiences with cCBT were well-established questionnaires but principally study-specific developed questionnaires. Indirect measures such as completion, take-up, and dropout rates, as well as reasons for take-up and dropout were less common. However, there is considerable discrepancy regarding the objective’s definition and operationalization.

**Figure 2:**
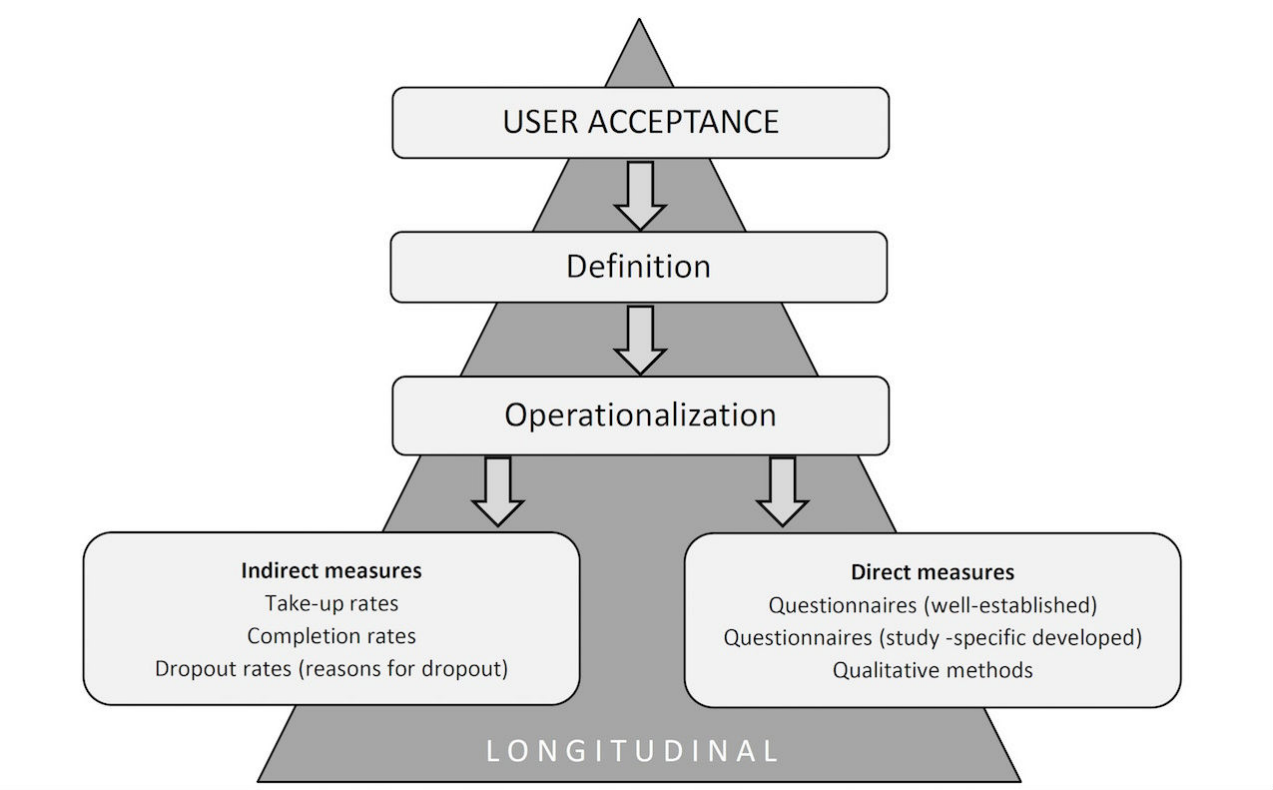
Recommended examination of user acceptance.

As can be seen in Figure 2, future research on user acceptance of cCBT should, therefore, include a theoretical framework and a definition of acceptance, adequate operationalization, and quantitative as well as qualitative data collection instruments that should be used in longitudinal and multidimensional approaches considering the stages of the process of acceptance.

The consideration of qualitative data is important since the accumulated material contains more details about the perspectives of trial participants than quantitative data does. Hence, in addition to take-up, completion, and dropout rates, it is important to learn about the reasons for take-up and dropout because one cannot be sure if discontinuing a treatment results from a negative attitude toward the treatment or other reasons such as those associated with research, technical, or personal circumstances.

A combination of quantitative and qualitative investigation examining expectations and experiences may prove beneficial. With the help of the juxtaposition of expectations and experiences, research on acceptance may fulfill its interpretation as a process in keeping with Kollmann’s [16] notion of acceptance, including the levels of attitude, action, and usage as well as Rogers’ [17] considerations regarding the five stages of the innovation-decision process. For this purpose, appropriate measures should be developed that are suitable for longitudinal studies. In general, the examination of user acceptance should be included alongside trials that focus basically on effectiveness.

In accordance with Kaltenthaler et al [21] and Waller and Gilbody [20], future research on user acceptance of innovative treatments such as cCBT should also include health care providers. Since physicians and therapists give therapy recommendations to their patients, it is important to learn more about their attitude toward cCBT.

## Abbreviations

ARM: Agnes-Davies Relationship Measure
AST: Acceptability of Self-Guided Treatment Questionnaire
BDI: Beck depression inventory
BL: baseline
CBT: cognitive behavioral therapy
cCBT: computerized cognitive behavioral therapy
CEQ: Credibility or Expectancy Questionnaire
CSQ-8: Client Satisfaction Questionnaire
FU: follow-up
iBA: Internet-based behavioral activation
iCBT: Internet-based cognitive behavioral therapy
IG: intervention group
MS: multiple sclerosis
PICO: Patient, problem, or population; Intervention; Comparison, control, or comparator; Outcome
PRISMA: Preferred Reporting Items for Systematic Reviews and Meta-Analyses
RCT: randomized controlled trial
SD: standard deviation
TAI: therapy attitude inventory
TAM: technology acceptance model
TAU: treatment-as-usual
VAS: visual analogue scale
ZUF-8: Fragebogen zur Patientenzufriedenheit (German version of CSQ-8)

## Appendix

Multimedia Appendix 1 Characteristics of the studies reviewed.

Multimedia Appendix 2 Measures and results of the studies reviewed.

Multimedia Appendix 3 Dropout and reasons for dropout of the studies reviewed.
