# Supplementary material for: User Acceptance of Computerized Cognitive Behavioral Therapy for Depression: Systematic Review
Source: J Med Internet Res. 2017 Sep 13;19(9):e309. doi: 10.2196/jmir.7662 (PMC5617907; doi:10.2196/jmir.7662)
Supplement: Multimedia Appendix 1 [file jmir_v19i9e309_app1.pdf]

Characteristics of the studies reviewed.

Author, year, country	Study design		Setting; recruitment	Sample[a]		
	Study type	Study duration (test-retest intervals)		n (total, intervention group)	Age, in years (mean [SD[b]], range)	Sex; female, male (% (n/N))
Ahmedani et al, 2015, United States [41]	Noncomparative (pilot feasibility study)	Pretreatment, posttreatment, FU[c]1 (after 2 weeks via telephone)	Clinic-based; referral by 2 physicians from a spine clinic	64	>49	67% (45/64), 33% (19/64)
					n=45 (70.3%)	
	Longitudinal				<50	
					n=19 (29.7%)	
Berger et al, 2011, Switzerland [25]	RCT[d]	Pretreatment, posttreatment (after 10 weeks), FU1 (at 6 months)	Community-based; newspaper articles and a TV interview	76	38.8 (14.0), 20-78	70% (53/76), 30% (23/76)
				(IG[e] I: n=25)		
	Longitudinal			(IG II: n=25)		
Berman et al, 2014, United States [31]	Noncomparative (feasibility study)	BL[f], midtreatment (after 4 weeks), study end point (after 10 weeks)	Community-based (university) and outpatient based; referral from the employee assistance program, a health coaching program, posters, and newspaper advertisements	29	53 (12.6)	79%, 21%
	Longitudinal					

Boeschoten et al, 2012, The Netherlands [42]	Noncomparative (pilot study)<br>Longitudinal	BL, postintervention	Clinic- and outpatient-based; clinical patient registry, advertisements in MS[g] newsletters, and direct referral by neurologists	44	45 (12)	77%, 23%
Burns et al, 2011, United States [43]	Noncomparative (single-arm field trial)<br>Longitudinal	BL, midtreatment (at week 4), posttreatment (at week 8)	Community-based; Web-based advertising venues	8	37.4 (12.2), 19-51	88% (7/8), 12% (1/8)
Cartreine et al, 2012, United States [32]	RCT (pilot feasibility study)<br>Longitudinal	BL, posttreatment 1 week (after the final ePST session), FU (4 weeks after completion)	Community-based; emailed information to persons listed on a registry of those interested in receiving information about clinical trials for depression	14 (7)	IG: 48.6 (10.2)	IG: 71% (5/7), 29% (2/7)
Choi et al, 2012, Australia [44]	RCT<br>Longitudinal	Pretreatment, posttreatment, FU (after 3 months)	Community-based; applicants applied online after reading details about the study and inclusion and exclusion criteria in the mass media	55 (25)	39.04 (11.68), 21-68	80% (44/55), 20% (11/55)

Danaher et al, 2013, United States [24]	Noncomparative (pilot feasibility trial)<br>Longitudinal	Pretest, posttest (after 3 months), FU (after 6 months)	Community- and clinic-based; birth records, nurse or health professional referrals, Web-based advertisements, and news stories to local university and hospital settings	53	31.9 (5.1)	100% (53/53), 0% (0/53)
de Graaf et al, 2009, The Netherlands [45]	RCT<br>Longitudinal	BL, FU1 (at 3 months), FU2 (at 9 months)	Community-based; recruitment in the general population by means of a large-scale Internet-based screening	200		
				(100)	IG: 44.3 (11.8)	IG: 52%, 48%
				(100)	IG + TAU[i]: 45.2 (10.9)	IG + TAU: 63%, 37%
Dear et al, 2013, Australia [30]	Noncomparative (single-group open trial)<br>Longitudinal	Pretreatment to posttreatment, posttreatment to FU (after 3 months)	Community-based; clinical research website set up for iCBT[h] trials	20	63.4 (5.08), 60-80	65% (13/20), 35% (7/20)
Dimidjian et al, 2014, United States [51]	Comparative (open trial)<br>Longitudinal	BL, posttreatment, FU1 (after 2.5 months),<br>FU2 (after 6 months)	Outpatient- and clinic-based; self-referral, referral by primary care or behavioral health clinicians, identification by electronic	200, (100)	IG: 47.40 (11.43)	IG: 73%, 27%

			medical records			
Geraedts et al, 2015, The Netherlands [46]	RCT<br>Longitudinal	BL, posttreatment (after 8 weeks)	Community-based (workplace); banners on the company's intranet, pamphlets, and posters	116	43 (8.9)	66.4% (77/116), 33.6% (39/116)
Gerhards et al, 2011, The Netherlands [33]	Qualitative study<br>Cross-sectional	Semistructured interviews	Community-based; interview participants were recruited from the cCBT[j] and cCBT+TAU groups of a previous trial	18	43.6 (14.5)	50% (9/18), 50% (9/18)
Hind et al, 2010, United Kingdom [28]	Qualitative study<br>Cross-sectional	Brief written feedback on a weekly evaluation sheet, brief semistructured telephone interviews in the days following the first session, depth face-to-face interviews after completion or withdrawal	Community-based (1 participant decided to undertake cCBT in the clinical research facility, the others worked on it at home); recruitment through letters to former patients of a MS clinic	17<br>(IG I: n=8)<br>(IG II: n=9)	46, 30-61	77% (13/17), 23% (4/17)
Høifødt et al, 2013, Norway [12]	RCT	BL, posttreatment, posttreatment (at 6 months)	Outpatient-based (primary care);	106 (52)	36.1 (11.3), 18-63	72.6% (77/106), 27.4% (29/106)

	Longitudinal		recruitment by written information provided by general practitioners and primary care nurses			
Kay-Lambkin et al, 2011, Australia [35]	RCT	BL, FU1 (after 3 months), FU2 (after 6 months), FU3 (after 12 months)	Study clinic–based; recruitment via a range of sources, most commonly via self-referral in response to TV interviews with the investigators or newspaper articles	97 (32)	35.37 (10.21)	54% (52/97), 46% (45/97)
	Longitudinal	The here reported results refer to the 3-month outcomes				
Knowles et al, 2015, United Kingdom [29]	Qualitative Study	Semistructured interviews	Outpatient-based (primary care); first 80 participants to complete their 4-month FU in the main trial were invited by letter to participate	36	51, 26-69	72% (26/36), 28% (10/36)
	Cross-sectional					
Kok et al, 2014, The Netherlands [47]	Noncomparative (subset of RCT)	BL, self-report questionnaires	Community- and outpatient-based; recruitment via media, general practitioners,	129	Assigned but did not finish module 1 (n=20): 42.1 (12.1)	80% (16/20), 20% (4/20)

	Longitudinal		and mental health services		Finished at least one module but not all 8 (n=45): 45.4 (10.4)	82% (37/45), 18% (8/45)
					Finished all 8 modules (n=64): 47.4 (10.7)	77% (49/64), 23% (15/64)
Lintvedt et al, 2013, Norway [48]	RCT<br>Longitudinal	Preintervention, postintervention (after 8 weeks)	Community-based (university); emailed screening survey to all registered students at the University and the University College of Tromsø	163 (81)	28.2 (7.4)	76.7% (125/163), 23.3% (38/163)
Lucassen et al, 2014, New Zealand [36]	Noncomparative (pilot feasibility trial)<br>Longitudinal	BL, postintervention, FU (after 3 months)	Community-based (school, organization); encouragement through youth-led organization for sexual minority youth and sexual minority media	21	16.5 (1.6), 13-19	48% (10/21), 52% (11/21)
McMurchie et al, 2013, United Kingdom [34]	Comparative<br>Longitudinal	BL, treatment end point (after 2 months), FU (after 3 months)	Outpatient-based; recruitment via older people's community	53 (33)	IG + TAU: 71.58 (4.43)	IG + TAU: 76% (25/33), 24% (8/33)

			mental health teams			
Merry et al, 2012, New Zealand [37]	RCT (randomized controlled noninferiority trial)<br>Longitudinal	BL, postintervention (after 2 months), FU (after 5 months)	Clinic- and outpatient-based (primary care), community-based (school); referral by clinicians, health care professionals, and school guidance counselors	187 (94)	IG: 15.55 (1.54)	IG: 63% (59/94), 37% (35/94)
O'Mahen et al, 2013, United Kingdom [49]	RCT<br>Longitudinal	BL, FU1 (after 15 weeks)	Community-based; advertisements on the "Netmums parenting website" and the Netmums newsletter	910 (462)	IG + TAU: 32.3 (4.7)	IG + TAU: 100% (462/462), 0% (0/462)
Perini et al, 2009, Australia [38]	RCT<br>Longitudinal	Pretreatment, midtreatment (after 4 weeks), posttreatment (after 1 week)	Community-based; recruitment via the website	45 (29)	49.29 (12.06), 19-85	78% (35/45), 22% (10/45)
Richards and Timulak, 2013, Ireland [26]	RCT<br>Longitudinal	BL, posttreatment (after 8 weeks)	Community-based (university); registered students at an Irish university with mild to moderate depressive	80<br>IG I: n=43<br>IG II: n=37	 IG I: 26.53, 20-50<br>IG II: 26.65, 19-59	 IG I: 58% (25/43), 42% (18/43)<br>IG II: 70% (26/37), 30% (11/37)

			symptoms as established by the BDI[k]-II			
Schneider et al, 2014, United Kingdom [39]	RCT<br>Longitudinal	BL, FU1 (after 6 weeks), FU2 (after 12 weeks)	Community-based (workplace); 3 large UK employers: 2 private enterprises + one public sector employer	637 (318)	IG: 42.2 (9.6)	IG: 55.3% (176/318), 42.8% (136/318)<br>(n=6 missing)
Sheeber et al, 2012, United States [40]	RCT (pilot trial)<br>Longitudinal	BL, T2 (14 weeks after BL), T3 (12 weeks after T2); (for persons assigned to Mom-Net, the T3 assessment was a FU, for persons initially assigned to delayed intervention or TAU T3 was a postassessment that enabled to examine response to the intervention)	Community-based (Head Start); invitation letters that Head Start staff gave to mothers	70<br>(IG I: n=35)<br>(IG II: n=35)	IG I: 31.1 (5.7)<br>IG II: 30.9 (7.0)	100% (70/70), 0% (0/70)
Stasiak et al, 2014, New Zealand [50]	RCT (a pilot double-blind ran-domized placebo controlled trial)<br>Longitudinal	BL, postintervention, FU1 (after 1 month)	Community-based (school); self-referral to school counselors	34 (17)	15.2 (1.5), 13-18	41% (14/34), 59% (20/34)
Titov et al, 2010, Australia [27]	RCT (randomized controlled noninferiority trial)<br>Longitudinal	BL, posttreatment (after 11 weeks), FU (after 4 months)	Community-based; information on the website	127	43 (12.86), 19-73	74% (94/127), 26% (33/127)

[a]All values are indicated as in studies provided.

[b]SD: standard deviation.

[c]FU: follow-up.

[d]RCT: randomized controlled trial.

[e]IG: intervention group.

[f]BL: baseline.

[g]MS: multiple sclerosis.

[h]iCBT: Internet-based cognitive behavioral therapy.

[i]TAU: treatment-as-usual.

[j]cCBT: computerized cognitive behavioral therapy.

[k]BDI: Beck depression inventory.
