# Supplementary material for: User Acceptance of Computerized Cognitive Behavioral Therapy for Depression: Systematic Review
Source: J Med Internet Res. 2017 Sep 13;19(9):e309. doi: 10.2196/jmir.7662 (PMC5617907; doi:10.2196/jmir.7662)
Supplement: Multimedia Appendix 2 [file jmir_v19i9e309_app2.pdf]

Table 4. Measures and results of the studies reviewed.

| Author, year, program | Object of investigation; measures | User satisfaction, experiences, and acceptability of treatment | Level of acceptance[a] (adjusted by the author) |
|---|---|---|---|
| Ahmedani et al, 2015 [41] | Acceptability | Participant ratings on the intervention were positive; over 75% of participants enjoyed working with the computer tablets, nearly 86% rated the patient and physician videos highly; almost 70% said participating in the intervention group (IG) got them thinking about their depression; the majority said being more likely to talk with their doctor about their depression (60.9%) and making changes in their daily lives to help with depression (60.9%) as a result of participating in this project; 75% stated that others would benefit "quite a lot" or "very much" from completing the intervention. | + |
| Internet-based cognitive behavioral therapy (iCBT) program (no name) | Participant acceptability ratings of the intervention were characterized along with items of interest in treatment seeking | | |
| Berger et al, 2011 [25] | Satisfaction | Participants reported a medium to high level of satisfaction in the active treatment arms; scores on satisfaction scale: 3.12 (standard deviation [SD] 0.44) for the guided self-help condition, thus falling between "somewhat" (3) and "very satisfied" (4); 2.86 (SD 0.53) for the unguided self-help group. | + |
| Deprexis (guided or unguided) | ZUF-8[b] (based on CSQ-8[c]) | | |
| Berman et al, 2014 [31] | Acceptability | Mean score for acceptability of the intervention remained stable from week 4 (mean=5.29, SD=1.1) to week 10 (mean=5.46, SD=1.3); on average, participants felt that ePST was acceptable as a stand-alone treatment for depression (indicating that they felt they did not need a clinician's input). | + |
| ePST | AST[d], including 16 statements that participants respond to on a 7-point Likert scale | | |
| Boeschoten et al, 2012 [42] | Satisfaction | Whereas 85% of the patients rated the quality of the intervention as good or excellent, 77.5% were | + |

| | | | |
|---|---|---|---|
| Computerized cognitive behavioral therapy (cCBT) program (no name) | CSQ-8; 10-point VAS[e] of their own devising (opinion about the website, support, and total intervention; higher scores=more satisfaction) | satisfied with the amount of help they had received; 75% thought the intervention had helped them to deal with their emotional problems; approximately 62% reported that the intervention had met their needs; 82.5% would recommend this kind of therapy to others; 60% would use the same intervention if they needed help again; CSQ-8: 23.6 (SD 4.8) for the total sample; regarding the VAS, support from the coach received the highest rank, that is, 7.7 (SD 1.2), the website scored 7.2 (SD 1.1), and the total intervention scored 7.2 (SD 1.3). | |
| Burns et al, 2011 [43] | Satisfaction | Treatment completers rated their satisfaction with the mobile phone in general by agreeing or disagreeing with the statement "I am satisfied with it"; their average rating was 5.71 (SD 1.38) on a scale rating from 1 (=strong disagreement) to 7 (=strong agreement); during coaching calls, 86% indicated that the intervention was helpful in understanding triggers for negative moods and increasing their ability to recognize and modify distressing behaviors and cognitions; participants' suggestions included lengthening the intervention and adding additional features such as a blog, messaging with coaches, or a recording tool to allow verbal elaboration on states when training by the phone. | + |
| Mobilyze! | Semistructured interview of their own devising, Web-based self-reports at each assessment, and information in coaching sessions for gathering participant feedback | | |
| Cartreine et al, 2012 [32] | Acceptability | Participants found ePST to be acceptable; of particular note were answers to the items "Doing problem-solving treatment using this program was acceptable to me" (mean=6.3, SD=1.1; median=7, range=4-7) and "I would feel comfortable using this program without a clinician's supervision" (mean=6.4, SD=0.8; median=7, range=5-7). | + |
| ePST | AST including 16 statements that the user responded to on a 7-point scale | | |
| Choi et al, 2012 [44] | Satisfaction | Moderate level of satisfaction with the program (overall satisfaction with program: 74% very satisfied, 33% neutral or somewhat satisfied, 4% | + |

| | | | |
|---|---|---|---|
| The Brighten Your Mood program | 7-item treatment satisfaction questionnaire enquiring about the acceptability of the modified Chinese depression treatment protocol based on the CEQ[f] | somewhat dissatisfied; satisfaction with quality of the treatment modules: 74% very satisfied, 17% neutral or somewhat satisfied, 9% somewhat dissatisfied); on a scale of 1 (=low level) to 10 (=high level of agreement), people rated the treatment as logical (mean=7.43, SD=1.90) and reported feeling confident that the treatment would be successful at teaching techniques for managing symptoms (mean=6.35, SD=2.10); participants would recommend this program to a friend with depression (mean=7.39, SD=1.95); 96% reported it was worth their time doing the program. | |
| Danaher et al, 2013 [24] | Satisfaction | Participants reported being quite satisfied with the features of the intervention (mean=3.3, SD=0.4); personal coach calls were rated as being helpful (mean=3.4, SD=0.9); comments on program satisfaction reflect positive feedback throughout with regard to the support by phone and positive aspects in doing something for one self, tasks broken down into steps, and being free in time management; personal coach calls were well received, by feeling someone was caring, helping remember to log in. | ++ |
| MomMood-Booster | 4-point scale (1=not at all satisfied, 4=very satisfied; not at all helpful to very helpful); open-ended questions about satisfaction | | |
| de Graaf et al, 2009 [45] | Acceptability | Participants rated the cCBT as acceptable in terms of expectancy, credibility, and pre- and posttreatment satisfaction: Scores on the CEQ were moderately high (Expectancy: cCBT mean=18.3, SD=4.2, cCBT+TAU mean=19.0, SD=4.8; Credibility: cCBT mean=18.8, SD=4.0, cCBT+TAU mean=19.2, SD=3.8); the majority of the participants expected that they would be less depressed after treatment (cCBT: n=33; cCBT+TAU: n=33) or that they would cope with their depression (cCBT: n=50; cCBT+TAU: n=44); most patients were satisfied with their treatment allocation (Yes=66 for cCBT, 71 for cCBT+TAU; Neutral=29 for cCBT, 25 for cCBT+TAU; No=5 for cCBT, 4 for cCBT+TAU). | + |
| Colour Your Life | Adapted Dutch version of the English CEQ; Expectancy question; Satisfaction with treatment allocation (1=yes, 2=neutral, 3=no); Evaluation questionnaire (7 statements to be rated on a 5-point scale, 1=completely agree, 5=completely disagree) | | |
| Dear et al, 2013 [30] | Satisfaction | Participants rated a high level of satisfaction; 82% said they would recommend the course to a friend | ++ |

| | | | |
|---|---|---|---|
| Managing Your Mood | Two questions regarding recommendation to a friend and the worthiness of time | and 82% reported that the doing the program was worth their time. | |
| Dimidjian et al, 2014 [51]<br>Mindful Mood Balance | Acceptability<br>Attendance, home practice completion | With regard to self-reported home mindfulness practice (assigned to be completed 6 days per week), within the full sample, mean weekly frequency of formal practice was 2.56 times (SD 2.15) and 55.80 min (SD 50.67) and for the 3-min breathing space practice was 8.91 times (SD 7.34) and 28.55 min (SD 24.13) per week; participants showed strong acceptance of the intervention and home practice, albeit at lower levels than have been reported for in-person groups; for completion rates see Table 3. | ? |
| Geraedts et al, 2015 [46]<br>Happy@-Work | Satisfaction<br>Internet Intervention Evaluation Questionnaire, containing quantitative and qualitative questions; participants grading the website, feedback, and the intervention on a scale from 1 to 10 and giving comments or suggestions for improvement; to identify reasons for dropout, an adapted version of the Internet Intervention Adherence Measure was used | Satisfaction with the intervention, feedback, and website was sufficient, with all grades above 7; the website was graded 7.4 (SD 0.9); the feedback was graded 7.7 (SD 1.3); the intervention was graded 7.4 (SD 1.2); needing a longer period of time to complete the intervention was reported most frequently, 76% reported that they would like to follow a Web-based intervention again in the future; for dropout rates and reasons see Table 3. | + |
| Gerhards et al, 2011 [33] | Experiences | Barriers and motivators experienced within cCBT were related to the course content and to contextual social, computer, and research aspects: | ~ |

| | | | |
|---|---|---|---|
| Colour Your Life | Semistructured interviews with open questions guided by a topic list | main barriers included experiencing a lack of identification with and applicability of cCBT, lack of support to adhere to the program or to gain a deeper understanding, and inadequate computer or Internet skills, equipment, or location; motivators included the opportunity to perform the therapy at your own time, pace, and place; adding support to cCBT was suggested as an improvement toward adherence and the course content. | |
| Hind et al, 2010 [28] | Acceptability | People felt that cCBT was a burden because of the physical and cognitive symptoms of multiple sclerosis or because it competed with other demands on their time; the absence of a human therapist meant that individuals felt cCBT was an isolating experience and that they had trouble defining suitable problems, setting goals, and applying CBT techniques; most felt that the program failed to (and needed to) acknowledge the role that an incurable condition played in their depression. | − |
| Beating the Blues or MoodGYM | Depth interviews | | |
| Høifødt et al, 2013 [12] | Acceptability, Satisfaction | Results are reported for IG and delayed-treatment control group (CG): overall satisfaction with treatment was high, with 89% giving the intervention as a whole a rating of 4 or 5; most participants indicated that they would recommend the combined intervention to a friend with a similar problem; the ratings of the intervention were positive but somewhat more moderate (between 50%-60%, giving clearly positive ratings to the benefit of the program, the usefulness of the exercises, and the relevance of the thematic content, and none rating the program as not useful or relevant); the benefit of the treatment sessions and the relationship with the therapist were rated positively by more than 90%. | + |
| MoodGYM | Nine questions to be rated on a 5-point scale (higher scores=greater satis-faction); the questions concerned their satisfaction with the intervention as a whole and various aspects of the self-help program and follow-up (FU) sessions | | |
| Kay-Lambkin et al, 2011 [35] | Acceptability | Take-up rates were high (97%); session attendance: mean=6.9; 63% (20 out of 32) attended an adequate dose of therapy (6 or more sessions); On average, alliance scores increased | + |

| | | | |
|---|---|---|---|
| SHADE | Treatment attendance measured by completion rates; therapeutic alliance measured by ARM[g] containing 28 self-report items to be rated on a 7-point Likert scale regarding client- and therapist-based domains and impressions of the client-therapist relationship | over the treatment period (mean [session 1 vs 5]=1.01, SD=2.48; mean [session 1 vs 10]=0.92, SD=1.85; mean [session 5 vs 10]=0.04, SD=1.21); for completion rates see Table 3. | |
| Knowles et al, 2015 [29] | Experiences, Acceptability | Four subthemes of acceptability: “flexibility,” “autonomy,” “relational,” and “connectedness” that illustrate positive and negative aspects of cCBT: “positive” (N=9; Beating the Blues: n=4, MoodGYM: n=5): patient controls when to use; supported autonomy—empowering, encourages self-determination; appreciate anonymity or reduced pressure of not being face-to-face; comforting—“always there”; “negative” (N=10; Beating the Blues: n=2, MoodGYM: n=8): “too flexible”—easy to avoid, difficult to sustain; enforced autonomy—too demanding, felt like “work”; lacks empathic response; isolating, enhances feeling of loneliness; “ambivalent” (N=17; Beating the Blues: n=7, MoodGYM: n=10 MoodGYM): appreciated flexibility but greater monitoring or FU needed to support use; interrupted autonomy—didactic, did not feel like it was user led, lacks personalization—too generic; disconnection from characters. | ~ |
| MoodGYM<br><br>Beating the Blues | Semistructured interviews that include questions to explore expressed preference and engagement | | |
| Kok et al, 2014 [47] | Acceptability | Most participants rated all modules as useful and easy; modules 4, 5, and especially 6 were rated as | + |

| | | | |
|---|---|---|---|
| Depression-free | Participant's evaluation after each module about the perceived usefulness (very useful—not at all useful), perceived difficulty (very easy—difficult); qualitative experiences of therapists and participants for describing difficulties with the modules | difficult; the evaluations on usefulness and difficulty of all modules were not associated with the number of finished modules (all *P* values >.05); a few participants reported that doing the intervention was helpful and easy to perform, some participants mentioned difficulties concerning specific contents (eg, drawing final conclusions). | |
| Lintvedt et al, 2013 [48]<br>MoodGYM + BluePages | Satisfaction<br>Series of items assessing participant's views about the usefulness of MoodGYM and BluePages, how easy the applications were to use, how much the participants felt they had learned, if they would recommend them to others, and if they had done something different because of the applications | 83.3% found the websites useful or very useful; 76.7% reported the websites were easy or very easy to understand; 83.3% reported that they learned something from the websites and 63.3% would probably use the websites again in the future; 90% would definitely or probably recommend the websites to others; 36.7% expressed changing their behavior because of the websites. | + |
| Lucassen et al, 2015 [36] | Acceptability | 80% indicated that they would recommend Rainbow SPARX to friends; 85% thought that the intervention would appeal to other young people; the content of the program that received the | ++ |

| | | | |
|---|---|---|---|
| Rainbow SPARX | Postintervention satisfaction questionnaire assessing items on Rainbow SPARX's appeal, usefulness, and likability using a 5-point Likert response format (5=very useful or really liked); completion rates | highest usefulness ratings (≥4) was "learning about depression" and "relax—slow breathing and muscle relaxation"; aspects of the program that received the highest likability ratings (≥4) were "You can learn things by yourself at your own pace," "It is different to talking to a doctor or counselor," "I could do it at home," "It comes with a notebook I can keep," and "It's made especially for young people"; for completion rates see Table 3. | |
| McMurchie et al, 2013 [34] | Acceptability | Take-up rate of 56.9% shows that cCBT is acceptable for at least half of the older people who participated in the program; for dropout rates see Table 3. | + |
| Beating the Blues | Take-up rates, dropout rates | | |
| Merry et al, 2012 [37] | Satisfaction | 95% believed that the type of support they received would appeal to other teenagers, 80.5% would recommend the treatment to their friends, 53.2% would have liked the sessions to stay the length they were, 44.3% wanted the sessions to be longer, and 61.5% reported that they completed all or most of the set challenges ("homework"). | ++ |
| SPARX | Self-designed self-report questionnaire gathering information about user satisfaction with the program, evaluating the features of the intervention, yes or no answers, and open-ended items | | |
| O'Mahen et al, 2013 [49] | Acceptability | Reasons for signing up for the course: wanting useful skills (60.6%), trusting Netmums site or name (37.9%), difficulties with access to CBT, issues around privacy, speaking face-to-face (29.5%), and fear of having their child taken away (15.2%); key acceptability endorsements: flexible and convenient delivery of the treatment ("I could do it in my own time": 78.8%, "It was emailed to my inbox every week": 63.5%, "It was free": 63.5%), helping women to "help myself" (55.8%); women noted struggling to keep up with the program ("I felt I couldn't keep up with it": | + |
| Postnatal Internet-based behavioral activation (iBA[h]) | Questionnaires about acceptability at two time points of measurement: (1) after confirming participation (items associated with participants' reasons for signing up for the intervention), (2) at | | |

| | | | |
|---|---|---|---|
| | the 15-week FU (questions about meeting or failing participants' expectations and possible improvements of the intervention) | 75.6%, "I felt overwhelmed with the weekly sessions": 34.1%, "It wasn't relevant to me or my situation": 14.6%); 48.9% did not know what they would have preferred to the course, 23.4% would have preferred more information, and 17% wished for something that related better to their situation. | |
| Perini et al, 2009 [38] | Satisfaction | Participants reported an acceptable level of satisfaction with the overall program; 82% reported being either very satisfied or mostly satisfied; 94% rated the quality of the treatment modules as excellent or good; 71% rated the quality of Internet correspondence with the therapist as excellent or good, whereas 29% rated it as satisfactory; the average participant rated the treatment as logical (8/10); they reported feeling confident that the treatment would be successful at teaching them techniques for managing their symptoms (7/10); they expressed a high level of confidence in recommending this treatment to a friend with depression (8/10). | ++ |
| The Sadness Program | Posttreatment questionnaire; ratings from 1 to 10 (10=high level of agreement) | | |
| Richards and Timulak, 2013 [26] | Satisfaction | Nonsignificant trend for the self-administered cCBT (sacCBT) group: they found the treatment easy to use and that the treatment would have lasting effects, more so than the therapist delivered cCBT (tdcCBT) group; the majority found the Web-based treatment helpful (sacCBT: 87%, tdcCBT: 90%); participants liked having self-control over the administration of the program; the sacCBT group reported an engaging and user-friendly treatment (less so for the other group), tdcCBT group most liked anonymity and liked the range of CBT techniques and strategies of the treatment; participants reported that the treatment could be complicated and impersonal and involved a lot of work; the tdcCBT group least liked the lack of deadlines, the sacCBT group disliked that the treatment at times did not match the needs of the user, technical difficulties in using the | + |
| Beating the Blues (guided or unguided) | Four questions to be rated from "agree very strongly" to "disagree very strongly" (use of the personal computer to access treatment, ease of use, lasting effects of the treatment, recommendation of cCBT to others, rating of the treatment's helpfulness); 2 qualitative questions | | |

| | | | |
|---|---|---|---|
| | (description of what participants most and least liked about the treatment) | program, and a possible irritating format of delivery. | |
| Schneider et al, 2014 [39]<br>MoodGYM | Acceptability<br>Questionnaire for judging the importance of 5 statements reflecting aspects of acceptability; open-ended statement regarding reasons to like or dislike help via the Internet; questions aiming at the relative acceptability of Web-based self-help compared with personal consultations | Strong agreement at baseline (BL) and FU1 with all of the following assertions (rated as “important” or “very important”): “I can use the computer at my own pace.” (BL: 89.9%, FU1: 90.8%); “using a computer is anonymous, I don't need to tell people about my problems.” (BL: 74.8%, FU1: 73.6%); “It is convenient for me to access help via the Internet and not to have to go to a health center or clinic.” (BL: 83%, FU1: 82.2%); “I can access help at any time that suits me.” (BL: 94%, FU1: 90%); “The computer will not criticize me.” (BL: 63.2%, FU1: 58.9%). A majority regarded Web-based self-help to be equally or more acceptable than seeing health care professionals face-to-face. | ++ |
| Sheeber et al, 2012 [40]<br>Mom-Net program | Satisfaction<br>Adapted version of the TAI[i] (ratings of the helpfulness of skills and materials, website, including ease of use, coach assistance, and program as a whole) | With regard to the program satisfaction, the mean ratings were above 4 on the 5-point Likert scale for skills, coach support, and general satisfaction across both conditions, indicating that the participants were highly satisfied with the intervention. | ++ |
| Stasiak et al, 2014 [50] | Acceptability | 55.5% liked the program, 56.6% rated it excellent or good, 66.7% would recommend it “as is” to other adolescents; participants identified five | + |

| | | | |
|---|---|---|---|
| The Journey | Brief satisfaction questionnaire of their own devising (perceived appeal, likes, dislikes, usefulness of specific features, and topics of the program); depth interview at the end of the study | features of the program as their favorite: “it was computer-based,” “showed me things I didn’t know about,” “I could use it at school,” “it was made for adolescents,” and “it talked about mental health”; the main identified weaknesses of the program were technical glitches, excessive amounts of reading, and perceived developmental inappropriateness (the program was thought to be more appealing to younger adults). | |
| Titov et al, 2010 [27] | Satisfaction | Acceptable level of satisfaction with the overall program (87% being either very satisfied or mostly satisfied, 13% neutral or somewhat dissatisfied, 0% very dissatisfied); 90% rated the quality of the treatment modules as excellent or good; 81% rated the quality of Internet correspondence with the clinician or technician as excellent or good, 14% rated it as satisfactory, 4% as unsatisfactory; the average participant rated the treatment as logical (8/10), they reported feeling confident that the treatment would be successful at teaching them techniques for managing their symptoms (8/10), they reported a high level of confidence in recommending this treatment to a friend with depression (8/10); no between treatment group differences were found in these items. | ++ |
| The Sadness Program (technician- or clinician-assisted) | Treatment satisfaction questionnaire (based on the CEQ) | | |

[a]Level of acceptance: ++ very high level of acceptance; + high level of acceptance; − moderate level of acceptance; − − low level of acceptance; ~ considerations of positive and negative aspects.

[b]ZUF-8: Fragebogen zur Patientenzufriedenheit, German version of CSQ-8 (high scores=greater satisfaction).

[c]CSQ-8: Client Satisfaction Questionnaire (maximum score of 32, higher scores=greater satisfaction).

[d]AST: Acceptability of Self-Guided Treatment (1=strong disagreement, 7=strong agreement; that the program is acceptable).

[e]VAS: visual analogue scale.

[f]CEQ: Credibility or Expectancy Questionnaire (high scores=greater satisfaction).

[g]ARM: Agnes-Davies Relationship Measure (higher scores=more positive perceptions of alliance).

[h]BA: Internet-based behavioral activation.

[i]TAI: therapy attitude inventory (higher scores=more positive experiences).
