# Supplementary material for: User Acceptance of Computerized Cognitive Behavioral Therapy for Depression: Systematic Review
Source: J Med Internet Res. 2017 Sep 13;19(9):e309. doi: 10.2196/jmir.7662 (PMC5617907; doi:10.2196/jmir.7662)
Supplement: Multimedia Appendix 3 [file jmir_v19i9e309_app3.pdf]

Dropout and reasons for dropout of the studies reviewed.

Author, year, country	Dropout and completion rates	Reasons for dropout in the intervention group
Ahmedani et al, 2015, United States [41]	Dropout: 6 participants did not complete the main intervention	Technical difficulties due to wireless Internet malfunctions and other software issues
Berger et al, 2011, Switzer-land [25]	Completion: Lessons completed; entire program completed:	Not reported
	IG[a] I: 6.8 (SD[b] 3.75); 9 (36%) participants	
	IG II: 8.52 (SD 2.86); 14 (56%) participants	
Berman et al, 2014, United States [31]	Completion of the entire program: 21	Not reported
	23 (87%) completed 4 and more sessions (=an adequate dose of ePST)	
Boeschoten et al, 2012, The Netherlands [42]	Completion of the entire program: 52%	24% computer-related problems; 14% lack of time; 24% stopping the intervention be-cause of psychosocial and environmental problems such as job loss, an ended relationship, and personal or family matters; 24% other reasons as MS[c]-related problems, the intervention not meeting someone's needs, wanting to finish the intervention in their own time
	Module 1: 91%, module 2: 70%, and module 3: 64%	
	(None of the patients dropped out between modules 4 and 5)	
Burns et al, 2011, United States [43]	Dropout: 1 participant dropped out in week 3	Technical problems using the phone and website
	Completion of the entire program: 7	
Cartreine et al, 2012, United States [32]	No dropout	None because of no dropout
	Completion of the entire program: 7	
Choi et al, 2012, Australia [44]	Completion of the entire program within 8 weeks: 17 (68%), 1 additional participant finished the remaining lesson within 2 days of the program ending	Not reported
	5.56 (SD 0.77)	

Danaher et al, 2013, United States [24]	Completion of the entire program: 46 (87%)	Not reported
de Graaf et al, 2009, The Netherlands [45]	Completion (n, IG; IG+TAU[d]):<br><br>Session 1: 72; 76, Session 2: 63; 60, Session 3: 52; 55, Session 4: 42; 50, Session 5: 36; 47, Session 6: 31; 39, Session 7: 25; 36, Session 8: 14; 26<br><br>IG: 4 (SD 3.0)<br><br>IG+TAU: 4.0 (SD 3.4)	Not reported
Dear et al, 2013, Australia [30]	Completion of the entire program (within 8 weeks): 16 (80%); the average number of lessons completed was 4.60 (SD 0.75)	1 participant (5%) formally withdrew during the program for medical reasons, which prevented further participation
Dimidjian et al, 2014, United States [51]	Dropout*:* 47 participants discontinued intervention<br><br>Completion of the entire program: 42 (42%), 53 (53%) completed at least four sessions	Increase in symptom severity, lack of interest or motivation, and time burden
Geraedts et al, 2015, The Netherlands [46]	Dropout: 29 participants dropped out of the intervention, 16 in their own request, and 13 because of inactivity on the website<br><br>Completion of the entire program: 32 (26.7%)<br><br>Lesson 1: 105 (90.5%), Lesson 2: 87 (75%), Lesson 3: 67 (57.8%), Lesson 4: 57 (49.1%), Lesson 5: 45 (38.8%)	Personal or family issues (main reason), lack of time, intervention took too much time to follow, and time to complete the assign-ments was too short
Gerhards et al, 2011, The Netherlands [33]	Dropout: Among the 10 noncompleters, 3 (16.7%) never started cCBT[e] or changed to another form of help, whereas 7 (38.9%) interviewees had started cCBT<br><br>Completion of the entire program: 8 (44.4%)	Among the 7 interviewees, 3 experienced cCBT as inconvenient and therefore changed to another form of help for depression symptoms, whereas 4 interviewees did not
Hind et al, 2010, United Kingdom [28]	Dropout: 6 participants (2 used intervention I, 4 used intervention II) did not complete the course of cCBT	4 reported competing priorities on their time, 1 stated the content as irrelevant, and 1 gave no reason

Høifødt et al 2013, Norway [12]	Dropout: Total nonadherence was 40%<br>Completion: 86% of participants that started treatment (n=50), completed 2 or more modules, 3.8 (SD 1.7)	No longer in need of treatment, did not find the treatment useful, received other treatment option, got worse, moved, lack of time
Kay-Lambkin et al, 2011, Australia [35]	Completion of the entire program: 15 (47%); Mean of session attendance: 6.9	Not reported
Knowles et al, 2015, United Kingdom [29]	Not reported	Not reported
Kok et al, 2014, The Netherlands [47]	Completion of the entire program: 64 (58.7%); finished at least one module: 109 (84.5%), 5.5 (SD 3.1)	Not reported
Lintvedt et al, 2013, Norway [48]	Completion: 32 (74.4%) participants reported that they had used at least some of the Internet websites; 27 participants completed on average 63% (3.1 modules) of MoodGYM	Not reported
Lucassen et al, 2014, New Zealand [36]	Completion of the entire program: 17 (81%)	Not reported
McMurchie et al, 2013, United Kingdom [34]	Dropout: After group allocation, 5 participants in the IG+TAU group didn't begin their first treatment session; 9 (27.3%) IG+TAU participants dropped out before the final session	Experiencing significant deteriorations in physical health, referral for face-to-face psychological therapy
Merry et al, 2012, New Zealand [37]	Completion of the entire program: 48 (60%), 69 participants (86%) completed at least four modules, 50 participants (62%) completed most or all of the homework challenges set	Technical glitches, lack of time; lack of interest, not finding the resource helpful; being physically unwell and unable to attend appointment
O'Mahen et al, 2013, United Kingdom [49]	Not reported	Not reported
Perini et al, 2009, Australia [38]	Completion of the entire program: 20 (74%)	Not reported

Richards and Timulak, 2013, Ireland [26]	Completion:	Not reported
	4.05 (SD 2.9); 4 completed <4 sessions, 4 completed >3 sessions, 7 completed all 8 sessions	
	3.97 (SD 2.2); 2 completed >4 sessions, 5 completed >3 sessions, 3 completed all 8 sessions	
Schneider et al, 2014, United Kingdom [39]	Dropout: At 6 weeks: 45%, at 12 weeks: 63%	Not reported
	Completion: Out of a possible 20: 8.35 (SD 6.76)	
Sheeber et al, 2012, United States [40]	Completion:	Not reported
	IG I: Completion of the entire program: 63% (mean=6.4)	
	IG II: Completion of the entire program: 49% (mean=6.0)	
Stasiak et al, 2014, New Zealand [50]	Completion of the entire program: 16 (94%)	Not reported
Titov et al, 2010, Australia [27]	Completion:	Not reported
	IG I: 80% completed all 6 lessons	
	IG II: 70% completed all 6 lessons	

[a]IG: intervention group.

[b]SD: standard deviation.

[c]MS: multiple sclerosis.

[d]TAU: treatment-as-usual.

[e]cCBT: computerized cognitive behavioral therapy.
